# Structure of the (Total) Transformation Monoids Under Rank N Generators

**DOI:** 10.12688/f1000research.173831.2

**Published:** 2026-04-30

**Authors:** Hala M. Sulaiman, Asawer Al-Aadhami

**Affiliations:** 1 Department of Administrative and Financial Affairs, University of Technology, Baghdad, Iraq; 2 Department of Mathematics, College of Science, University of Baghdad, Baghdad, Baghdad Governorate, Iraq

**Keywords:** A finite group, Semigroup, Wreath product, A free (left) G-act, Total transformation semigroup, Endomorphism monoid, Semigroup morphism.

## Abstract

Throughout this paper our study focuses on transformation semigroups. These kinds of semigroups are the corner stone of semigroup theory. This is because every semigroup is isomorphic to transformation semigroup. The (total) transformation monoid $T_{X_{n}}$ on a finite set $X_{n}=\left\{1,2,\ldots ,n\right\}$ where n≥0 , n∈Z , is a semigroup of mapping that takes a set $X_{n}$ into itself, under the operation of composition of mapping with identity $I_{X_{n}}$ . In this paper, we use an algebraic method for considering the monoid $T_{{\left(\mathrm{Fl}\right)}_{n}\left(G\right)}$ , where an independence algebra ${\left(\mathrm{Fl}\right)}_{n}\left(G\right)$ is a disjointed union of sets of the form $Gx_{i}\;$ for all 1 ≤i≤n. Firstly, particular attention is paid to find the isomorphism between $T_{{\left(\mathrm{Fl}\right)}_{n}\left(G\right)}$ and the endomorphism monoid $\mathrm{End}{\left(F\ell \right)}_{n}\left(G\right).$ Secondly, the embeddedness of $T_{{\left(\mathrm{Fl}\right)}_{n}\left(G\right)}$ in (full) wreath product of $T_{n}$ by $G^{n}$ has been found. Finally, the description of Green’s relation of $T_{{\left(\mathrm{Fl}\right)}_{n}\left(G\right)}$ has been provided.

## 1. Introduction

In this paper G will be a finite monoid (group) and ${\left(F\ell \right)}_{n}\left(G\right)$ be a free (left)- G -act of rank n . It is clear that ${\left(F\ell \right)}_{n}\left(G\right)\;$ is an independence algebra, ^1^ which is a class of universal algebras of rank n generators that includes free (left)- G -acts for a finite group G. For a positive integer n , we write the (total) transformation $T_{X_{n}}$ to be $T_{n}$ which is the monoid of n degree, under the operation of a compositions of functions, such that for all $\sigma ,\tau \;\in \;T_{n}$ and $x\;\in \;X_{n}$ we have x(σ∘τ)=x(στ)=(xσ)τ. As well as, it is a set that maps every transformations into itself (i.e., $X_{n}⟶X_{n}$ , ∀n≥0,n∈Z) . Clearly, if n=0, then $X_{n}=\emptyset ,$ and $T_{0}$ becomes a monoid that consists of only one map, which is the (empty) map.

All transformations of this paper are composed from left to right, so for any $\sigma \;\in \;T_{n}$ and for any $i\;\in \;X_{n},$ then iσ will be written as the image of i , and for any $\sigma \;\in \;T_{n}$ , we define Im{σ}={iσ:i∈Xn} and $\mathrm{Ker}\left\{\sigma \right\}=\left\{\left(i,j\right)\;\in \;X_{n}\times X_{n}:\mathrm{i\sigma}=\mathrm{j\sigma}\right\}$ and ℘(σ)=rank{σ}=|Im{σ}|. A number of authors appeared in ^2– 9^ obtained and described various transformation semigroups, such as the description of the singular transformation $\mathrm{Sn}{ℊ}_{n},$ ^10^ and they study the structure of the partial endomorphism monoids of the independence algebra like a free acts and other set such as vector spaces and modules. When G is a finite monoids the endomorphism monoids of free (left)- G -acts played a major part of semigroup theory, and it can be found in. ^11^

It has long been known that set of the endomorphism monoid of the free (left)- G -acts of rank n is isomorphic to the (full) wreath product $G{≀}_{n}T_{n},$ ^12, 13^ or, more generally $S{≀}_{n}T_{n}$ for arbitrary semigroup S. Clearly, if G={e} , then $\;G{≀}_{n}T_{n}≅$ $T_{n}$ . Where *S* is a finite semigroup, the (total) transformation monoid $T_{{\left(F\ell \right)}_{n}\left(S\right)}$ of a free (left) S− act of rank n has been described in, ^12^ for further information we recommend the reader see. ^14– 21^ Through this work, the discerption of $T_{{\left(F\ell \right)}_{n}\left(M\right)}$ , has established in particular when M is a finite group, and many properties of this concepts have been studied such as the isomorphic of $T_{{\left(F\ell \right)}_{n}\left(M\right)}$ to $\mathrm{End}{\left(F\ell \right)}_{n}\left(M\right)$ and the embeddedness of it in the (total) wreath product $M{≀}_{n}T_{n}$ . Furthermore, in the last section of this work the discerption of Green’s relation of $T_{{\left(F\ell \right)}_{n}\left(M\right)}$ has been found.

## 2. Preliminaries

Definition 2.1: ^12^ A mapping σ from a monoid A to a monoid B is named (morphism) if

1- ( $a\bar{a}$ ) $\sigma =\left(a\right)\sigma \left(\bar{a}\right)\sigma ,$ for all a , $\;\bar{a}\;\in \;A$ ;
2- ( $1_{A})\sigma =1_{B}.$

Definition 2.2: ^12^ Let B be a non-empty set and A be a monoid, then B is refed to a (left)- A -act if there is a mapping α:A×B⟶B, defined by (a,b)α=ab , such the following conditions are satisfied for all b∈B and a,r∈A :

1 -
a(rb)=(ar)b .
2 -
$1_{A}b=b$ .

Definition 2.3: ^12^ Suppose that Y and T are two (left)- A -acts, then a mapping α:Y⟶T is named an A - acts morphism if (ay)α=a(yα) , ∀a∈A, y∈Y .

Definition 2.4: ^12, 13^ The (total) transformation semigroup on a non-empty set X is denoted by $\;T_{X}=\left\{\sigma |\sigma :X\to X\right\}.$ If n≥0 , such that n∈Z , where $X=X_{n}=\left\{1,2,\ldots ,n\right\},$ we write $\;T_{X}$ to be $\;T_{n}$ . With respect to the semigroup operation of composition that x(δ∘γ)=x ( δγ ) =(xδ)γ, for all x∈X and $\delta ,\gamma \;\in \;T_{x}$ . Clearly, $\;T_{n}$ is monoid with identity transformation $\;I_{X}=\left(\begin{array}{ccc} 1 & 2\ldots & n\\ 1 & 2\ldots & n \end{array}\right)$ .

Remark 2.5: ^12, 13^

1 - If $\sigma \;\in \;T_{n}$ , n≥0 , n∈Z, then σ can be written as $\sigma =\left(\begin{array}{ccc} 1 & \ldots & n\\ 1\sigma & \ldots & \mathrm{n\sigma} \end{array}\right)$ .
2 - For any $\sigma \;\in \;T_{n},$ the second row of σ is not a permutation for the first row. Since not all element σ∈ $T_{n}\;$ is injective.
3 - The number of elements of $\;T_{n}$ is equal to $n^{n}$ .

Recall that, if S is a semigroup and if t∈S , the (principal) left ideal generated by t is the smallest left ideal of S containing t , and it is denoted by $S^{1}t$ . Dually, for ${\mathrm{tS}}^{1}$ . ^22^

Definition 2.6: ^22^ Let S be any semigroup, the Green’s preorder binary relations are defined as follows:

1.
$s\;\leq_{L}\;t$ if and only if $s\;\in \;S^{1}t$ .
2.
$s\;\leq_{R}\;t$ if and only if $s\;\in \;{\mathrm{tS}}^{1}$ .
3.
$s\;\leq_{J}\;t$ if and only if $s\;\in \;S^{1}{\mathrm{tS}}^{1}.$

From the above definition we can define the Green’s relations by:

Definition 2.7: ^22^ Let S be any semigroup, the Green’s relations are defined by:

1.
$R=\;\leq_{R}\;\cap \;\geq_{R}.$

2.
$L=\;\leq_{L}\;\cap \;\geq_{L}.$

3.
$J=\;\leq_{J}\;\cap \;\geq_{J}.$

4.
H=R∩L.

5.
D=R∘L .

Clearly, if S is finite, then D=J. So, the above definition can be translated as follows:

Definition 2.8: ^22^ Let S be a finite semigroup, the Green’s relations can be defined as:

1.
sLt if and only if $S^{1}s=S^{1}t.$

2.
sRt if and only if ${\mathrm{sS}}^{1}={\mathrm{tS}}^{1}.$

3.
sJt if and only if $S^{1}{\mathrm{sS}}^{1}=S^{1}{\mathrm{tS}}^{1}.$

## 3. Free (left)- G -act structure ${\left(F\ell \right)}_{n}\left(G\right)$

This section is devoted to give an explicit description of the set of the free (left)- G -acts, when G is a finite group.

Definition 3.1: ^12, 22^ Let G be a finite group and X be a non-empty set, then ${\left(F\ell \right)}_{X}\left(G\right)$ is known as a free (left)- G -acts on X if:

1 - For any mapping $\sigma :X⟶{\left(F\ell \right)}_{X}\left(G\right);$

2 - For all G -acts Y and every map δ:X⟶Y, there exists a unique morphism $\;\phi :{\left(F\ell \right)}_{X}\left(G\right)⟶Y$ such that the following diagram is commute:

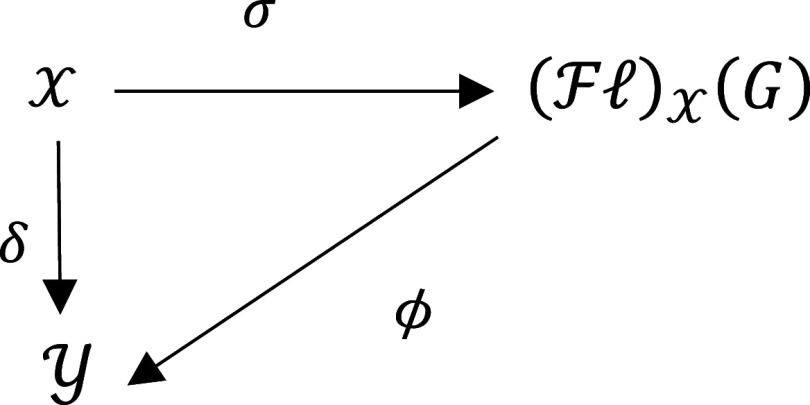

If $X=X_{n}=\left\{1,2,\ldots ,n\right\}$ where n≥0 and n∈Z, the free (left)- G -act can be written as ${\left(F\ell \right)}_{n}\left(G\right)$ .

It is clear that ${\left(F\ell \right)}_{n}\left(G\right)$ consists elements of the form gx, where g∈G and x∈X i.e., $\;{\left(F\ell \right)}_{n}\left(G\right)={\dot{\cup }}_{i=1}^{i=n}Gx_{i},$ such that $Gx_{i}=\left\{gx_{i}:g\;\in \;G,1\;\leq \;i\;\leq \;n,x_{i}\;\in \;X\right\}.$ ^1, 12^ It is well-known that the set of all the morphisms σ:B⟶B is called the endomorphism monoid, and it is denoted by End(B). Therefore, the set of all morphisms from the free (left)- G -acts into itself is an endomorphism monoid and it is denoted by $\mathrm{End}\;{\left(F\ell \right)}_{n}\left(G\right)$ such that $\mathrm{End}\;{\left(F\ell \right)}_{n}\left(G\right)=\{\sigma \;|\sigma :{\left(F\ell \right)}_{n}\left(G\right)⟶{\left(F\ell \right)}_{n}\left(G\right)$ ; and σ is a G− morphism}. If $\sigma \;\in \;\mathrm{End}\;{\left(F\ell \right)}_{n}\left(G\right)$ , we define σ to be $x_{j}\sigma =g_{j}^{\sigma }x_{j\overset{´}{\sigma },}$ where 1 ≤j≤n , $\overset{´}{\sigma }\;\in \;T_{n}$ , and $\;g_{j}^{\sigma }\;\in \;G^{n}$ . As σ is a G− morphism and for g∈G and 1 ≤j≤n we have $\left(gx_{j}\right)\sigma =g\left(x_{j}\sigma \right)=g\left(g_{j}^{\sigma }x_{j\overset{´}{\sigma }}\right)$ .

## 4. Properties of the (full) wreath product multiplication

The (full) wreath product multiplication in semigroup theory is a multiplication comes from a semidirect product between two semigroups. The next definition gives an abstract construction of the (full) wreath product multiplication.

Definition 4.1: ^12, 13^ Let G be a finite group and $T_{n}$ be the (total) transformation on $X_{n}$ , where n≥0,n∈Z.

Then, the (full) wreath product of $G^{n}\;$ by $\;T_{n}$ is

$$\left(g_{1}^{\delta },\ldots ,g_{n}^{\delta },\delta \right)\left(g_{1}^{\sigma },\ldots ,g_{n}^{\sigma },\sigma \right)=\left(g_{1}^{\delta }g_{1\delta }^{\sigma },\ldots ,g_{n}^{\delta }g_{\mathrm{n\delta}}^{\sigma },\delta \sigma \right),$$

where $\delta ,\sigma \;\in \;T_{n}$ and $g_{j}^{\delta },g_{j}^{\sigma }\;\in \;G^{n}.$ Under this multiplication, the set ( $G^{n}\times T_{n})$ is a monoid which is denoted by $G{≀}_{n}T_{n}$ , with identity $\;\left(e,e,\ldots ,e_{n},I_{n}\right),$ when $I_{n}\;$ is the identity of $T_{n}$ .

## 5. The structure of semigroup $\;T_{{\left(F\ell \right)}_{n}\left(G\right)}$

This section is devoted to describe the set $\;T_{{\left(F\ell \right)}_{n}\left(G\right)}$ , when ${\left(F\ell \right)}_{n}\left(G\right)$ is an independence algebra.

### 5.1 The monoids $T_{Y}$ and $T_{Y}$

Suppose ***B*** be an algebra and *B* be a universe of ***B***, ^1, 12, 13^ The sets $T_{B}=\left\{\sigma |\sigma :B⟶B,\forall \sigma \;\in \;T_{B}\right\}$ , and $T_{B}=\{\tau |\tau :B⟶B,$ such that τ is a morphism $,\forall \tau \;\in \;T_{B}$ } are monoids. Furthermore, $T_{B}$ is submonoid of $T_{B},$ this result has been proved by Al-Aadhami in, ^12^ and this can be shown by the following lemma:

Lemma 5.1.1: The semigroup $T_{B}\;$ is a submonoid of $T_{B}.$

Proof: See. ^12^

### 5.2 The monoid $\;T_{{\left(F\ell \right)}_{n}\left(G\right)}$

In this subsection we give an explicit description to the (total) transformation monoid $\;T_{{\left(F\ell \right)}_{n}\left(G\right)}$ .

Definition 5.2.1: The set of all morphisms can be defined as $\;T_{{\left(F\ell \right)}_{n}\left(G\right)}=\{\;\sigma \;|\sigma :{\left(F\ell \right)}_{n}\left(G\right)⟶{\left(F\ell \right)}_{n}\left(G\right)$ , such that σ is morphism} which is a monoid under the composition of mapping. If γ∈ $T_{{\left(F\ell \right)}_{n}\left(G\right)},$ then γ can be expressed as:

$$\gamma =\left(\begin{array}{ccc} x_{i_{1}} & \ldots & x_{i_{m}}\\ \;{ℊ}_{i_{1}}^{\gamma }x_{i_{1}\overset{´}{\gamma }}\; & \ldots & \;{ℊ}_{i_{m}}^{\gamma }x_{i_{m}\overset{´}{\gamma }} \end{array}\right).$$

Where $\overset{´}{\gamma }\;\in \;T_{n},{ℊ}_{i_{1}}^{\gamma },\ldots ,{ℊ}_{i_{m}}^{\gamma }\;\in \;G$ such that 1 $\;\leq \;i_{1}<\ldots <i_{m}\;\leq \;n,m\;\geq \;0$ and $x_{i_{k}}\gamma ={ℊ}_{i_{k}}^{\gamma }x_{i_{k}\overset{´}{\gamma }}$ . Notice, for every selection of $\overset{´}{\sigma }\;\in \;T_{n}$ with $\mathrm{Dom}\;\left(\overset{´}{\sigma }\right)=\left\{\ell_{1},\ldots .,\ell_{r}\right\}$ for $1\;\leq \;\ell_{1}<\ldots <\ell_{r}\;\leq \;n,r\;\geq \;0$ and ${ℊ}_{\ell_{1}}^{\sigma },\ldots ,{ℊ}_{\ell_{r}}^{\sigma }\;\in \;G$ this gives $\sigma =\left(\begin{array}{ccc} x_{\ell_{1}} & \ldots & x_{\ell_{r}}\\ \;{ℊ}_{\ell_{1}}^{\sigma }x_{\ell_{1\overset{´}{\sigma }}}\; & \ldots & {ℊ}_{\ell_{r}}^{\sigma }x_{\ell_{r\overset{´}{\sigma }}} \end{array}\right)\;\in \;T_{{\left(F\ell \right)}_{n}\left(G\right)}$ .

Clearly, where G={e}, i.e., ( G is a trivial set), then $\;T_{{\left(F\ell \right)}_{n}\left(G\right)}$ will be isomorphic to $T_{n}$ .

The next theorem is analogous to Lemma 4.1. ^12^ However, in the following theorem a semigroup S is assumed to be a finite group G.

Theorem 5.2.2: If n≥0 , n∈Z, then $\;T_{{\left(F\ell \right)}_{n}\left(G\right)}$ $≅\mathrm{End}{\left(F\ell \right)}_{n}\left(G\right).$

Proof: Suppose σ be a mapping such that $\sigma :T_{{\left(F\ell \right)}_{n}\left(G\right)}⟶\mathrm{End}{\left(F\ell \right)}_{n}\left(G\right)$ defined by $\lambda \sigma =\bar{\lambda }$ when $\left(gx_{i}\right)\bar{\lambda }=\left(gx_{i}\right)\lambda ,$ for all g∈G. We must show that $\bar{\lambda }$ is a G -acts morphism, i.e., we want to show that $g\left(b\bar{\lambda }\right)=\left(\mathrm{gb}\right)\bar{\lambda },$ for all g∈G and $b\;\in \;{\left(F\ell \right)}_{n}\left(G\right)$ . Let $b=kx_{i}$ . Clearly, an element $x_{i}\;\in \;\mathrm{Dom}\left(\lambda \right)\;$ if and only if $kx_{i}\;\in \;\mathrm{Dom}\left(\lambda \right)$ for all k∈G. Where $kx_{i}\;\in \;\mathrm{Dom}\left(\lambda \right)$ , that means for all g∈G , $\mathrm{gk}x_{i}\;\in \;\mathrm{Dom}\left(\lambda \right)\;$ then $\left(\mathrm{gk}x_{i}\right)\bar{\lambda }=\left(\mathrm{gk}x_{i}\right)\lambda =g\left(\left(kx_{i}\right)\lambda \right)=g\left(\left(kx_{i}\right)\bar{\lambda }\right),$ since λ is a G -acts morphism.

To show σ is injective. Assume that λσ=γσ , then for any $gx_{i}\;\in \;{\left(F\ell \right)}_{n}\left(G\right)$ obtaining ( $gx_{i})\lambda \sigma =\left(gx_{i}\right)\gamma \sigma ,$ ∀i. Notice that, ( $gx_{i})\lambda =\left(gx_{i}\right)\bar{\lambda }=\left(gx_{i}\right)\lambda \sigma =\left(gx_{i}\right)\gamma \sigma =\left(gx_{i}\right)\bar{\gamma }=\left(gx_{i}\right)\gamma$ , so λ=γ , as required.

To prove σ is onto. Let $\bar{\beta }\;\in \;\mathrm{End}{\left(F\ell \right)}_{n}\left(G\right)$ and let $\beta \;\in \;T_{{\left(F\ell \right)}_{n}\left(G\right)}$ defined from ${\left(F\ell \right)}_{n}\left(G\right)$ to ${\left(F\ell \right)}_{n}\left(G\right)$ by

$\left(gx_{i}\right)\beta =\left(gx_{i}\right)\bar{\beta }$ , for each $gx_{i}\;\in \;{\left(F\ell \right)}_{n}\left(G\right).$ As $\beta \;\in \;T_{{\left(F\ell \right)}_{n}\left(G\right)}\;$ that implies β is a G -acts morphism, therefore for some $gx_{i}\;\in \;\mathrm{Dom}\left(\beta \right)={\left(F\ell \right)}_{n}\left(G\right)$ and k∈G , we have $k\left(\left(gx_{i}\right)\beta \right)=k\left(\left(gx_{i}\right)\bar{\beta }\right)=\left(\mathrm{kg}x_{i}\right)\bar{\beta }=\left(\mathrm{kg}x_{i}\right)\beta$ .

Since, $\left(gx_{i}\right)\beta =\left(gx_{i}\right)\bar{\beta }=\left(gx_{i}\right)\beta \sigma$ , for all $gx_{i}\;\in \;{\left(F\ell \right)}_{n}\left(G\right)$ , g∈G. Therefore, $\beta \sigma =\bar{\beta }$ , that gives σ is onto.

To prove σ is a homomorphism. Let $\delta ,\gamma \;\in \;T_{{\left(F\ell \right)}_{n}\left(G\right)}$ .We need (δγ)σ=δσγσ . From the definition of σ and from our assumption we have $\;\left(gx_{i}\right)\left(\delta \gamma \right)\sigma =\left(gx_{i}\right)\bar{\delta \gamma }=\left(gx_{i}\right)\delta \gamma$ ∀i . Conversely, $\left(gx_{i}\right)\left(\text{δσγσ}\right)=\left(gx_{i}\right)\bar{\delta }\bar{\gamma }$ .

Obviously, $\bar{\delta ,}\bar{\gamma }\;\in \;\mathrm{End}{\left(F\ell \right)}_{n}\left(G\right)$ , so $\bar{\delta ,}\;$ and $\bar{\gamma }\;$ are a G -acts morphisms. Then, $\left(gx_{i}\right)\bar{\delta }\bar{\gamma }=\left(\left(gx_{i}\right)\bar{\delta }\right)\bar{\gamma }=\left(\left(gx_{i}\right)\delta \right)\bar{\gamma }=\left(\left(gx_{i}\right)\delta \right)\gamma =\left(gx_{i}\right)\delta \gamma =\left(gx_{i}\right)\bar{\delta \gamma }$ . So, $\left(\delta \gamma \right)\sigma =\bar{\delta \gamma }=\bar{\delta }\bar{\gamma }=\delta \gamma$ , and this complete the proof.□

Theorem 5.2.3: For all n≥0, n∈Z, $\;T_{{\left(F\ell \right)}_{n}\left(G\right)}$ $↪G{≀}_{n}T_{n}.$

Proof: Assume that $\sigma \;\in \;T_{{\left(F\ell \right)}_{n}\left(G\right)},$ then σ can be written as $=\left(\begin{array}{ccc} x_{i_{1}} & \ldots & x_{i_{m}}\\ \;g_{i_{1}}^{\sigma }x_{i_{1}\overset{´}{\sigma }}\; & \ldots & \;g_{i_{m}}^{\sigma }x_{i_{m}\overset{´}{\sigma }} \end{array}\right)$ , such that $\overset{´}{\sigma }\;\in \;T_{n},g_{i_{1}}^{\sigma },\ldots ,g_{i_{m}}^{\sigma }\;\in \;G$ for 1 $\;\leq \;i_{1}<\ldots <i_{m}\;\leq \;n,m\;\geq \;0$ . Let $\alpha :T_{{\left(F\ell \right)}_{n}\left(G\right)}⟶G{≀}_{n}T_{n}\;$ defined by $\mu \alpha =\left(g_{1}^{\mu },\ldots ,g_{n}^{\mu },\overset{´}{\mu }\right)$ . In order to prove α is embedding, we have to prove α is one to one and a homomorphism map. Let $\;\delta ,\gamma \;\in \;T_{{\left(F\ell \right)}_{n}\left(G\right)}$ , we need show ( δγ ) α=δαγα . Now, $\text{δαγα}=\left(g_{1}^{\delta },\ldots ,g_{n}^{\delta },\overset{´}{\delta }\right)\left(g_{1}^{\gamma },\ldots ,g_{n}^{\gamma },\overset{´}{\gamma }\right)=\left(g_{1}^{\delta }g_{1\overset{´}{\delta }}^{\gamma },\ldots ,g_{n}^{\delta }g_{n\overset{´}{\delta }}^{\gamma },\overset{´}{\delta }\overset{´}{\gamma }\right).$ The other side gives ( δγ ) $\;\alpha =\left(g_{1}^{\delta \gamma },\ldots ,g_{n}^{\delta \gamma },{\left(\delta \gamma \right)}^{\prime }\;\right)$ . As $\delta ,\gamma \;\in \;T_{{\left(F\ell \right)}_{n}\left(G\right)}$ then δ,γ are G -acts morphisms such that they take $\;T_{{\left(F\ell \right)}_{n}\left(G\right)}$ to itself. Hence, $\left(x_{i}\right)\delta \gamma =\left(x_{i}\delta \right)\gamma =\left(g_{i}^{\delta }x_{i\overset{´}{\delta }}\right)\gamma =g_{i}^{\delta }\left(x_{i\overset{´}{\delta }}\gamma \right)=g_{i}^{\delta }g_{i\overset{´}{\delta }}^{\gamma }x_{i\overset{´}{\delta }\overset{´}{\gamma }}$ ∀i, and ( $x_{i})\delta \gamma =g_{i}^{\delta \gamma }x_{i\left(\delta \gamma \right)\prime }$ . From that, ∀i we get $g_{i}^{\delta \gamma }x_{i\left(\delta \gamma \right)\prime }=g_{i}^{\delta }g_{i\overset{´}{\delta }}^{\gamma }x_{i\overset{´}{\delta }\overset{´}{\gamma }}$ , so we obtain $g_{i}^{\delta \gamma }=g_{i}^{\delta }g_{i\overset{´}{\delta }}^{\gamma }$ and ${\left(\delta \gamma \right)}^{\prime }=\overset{´}{\delta }\overset{´}{\gamma }$ . This implies, α is homomorphism.

To prove α is one-to-one. Let $\delta ,\gamma \;\in \;T_{{\left(F\ell \right)}_{n}\left(G\right)}\;$ such that δα=γα that leads to $\left(g_{1}^{\delta },\ldots ,g_{n}^{\delta },\overset{´}{\delta }\right)=\left(g_{1}^{\gamma },\ldots ,g_{n}^{\gamma },\overset{´}{\gamma }\right)$ .

Then for any $gx_{i}\;\in \;{\left(F\ell \right)}_{n}\left(G\right)$ , we have $\left(gx_{i}\right)\delta =g\left(x_{i}\delta \right)=g\left(g_{i}^{\delta }x_{i\overset{´}{\delta }}\right)=g\left(g_{i}^{\gamma }x_{i\overset{´}{\gamma }}\right)=g\left(x_{i}\gamma \right)=\left(gx_{i}\right)\gamma$ , and

then δ=γ and α is one-to-one.□

## 6. Green’s relations properties of $\;T_{{\left(F\ell \right)}_{n}\left(G\right)}$

Where B is an independence algebra V. Gould, ^1^ gave the characterization of Green’s relations on End(B) , after that A. Al-Aadhami, ^12^ described the Green’s relations on $\mathrm{End}\;{\left(F\ell \right)}_{n}\left(S\right)$ , where S is a finite semigroup. In this section the characterization of Green’s relations on $\;T_{{\left(F\ell \right)}_{n}\left(G\right)}$ have been illustrated.

Theorem 6.1: For all $\;\sigma ,\tau \;\in \;T_{{\left(F\ell \right)}_{n}\left(G\right)}$ , we get the following identities:

I.
$\sigma \;\leq_{L}\;\tau \;$ if and only if Im{σ}⊆Im{τ}.

II.
$\sigma \;\leq_{R}\;\tau \;$ if and only if Ker{τ}⊆Ker{σ}.

III.
℘(στ)≤℘(σ),℘(τ).

Proof:

I.
⟹) Let $\sigma \;\leq_{L}\;\tau$ in $\;T_{{\left(F\ell \right)}_{n}\left(G\right)}$ that implies σ=δτ for some δ∈ $\;T_{{\left(F\ell \right)}_{n}\left(G\right)}\;$ and so by, ^12^ [Proposition 5.5] we have Im{σ}=Im{δτ}⊆Im{τ} , and hence Im{σ}⊆Im{τ}.

⇐) Assume that Im{σ}⊆Im{τ} , so for all j∈{1,2,…,n} , we get xjσ∈Im{σ}⊆Im{τ}.

  If we select $b_{j}\;\in \;{\left(F\ell \right)}_{n}\left(G\right)$ such that $x_{j}\sigma =b_{j}\tau$ , then by defining υ∈ $\;T_{{\left(F\ell \right)}_{n}\left(G\right)}$ such that $x_{j}\upsilon =b_{j}$ for j∈{1,2,…,n}. It is clear that $x_{j}\upsilon \tau =b_{j}\tau =x_{j}\sigma \;$ and therefore we can obtain υτ=σ that means $\sigma \;\leq_{L}\;\tau .$

II.
⟹ (Where $\sigma \;\leq_{R}\;\tau$ in $\;T_{{\left(F\ell \right)}_{n}\left(G\right)}$ that implies σ=τλ for any $\lambda \;\in \;T_{{\left(F\ell \right)}_{n}\left(G\right)}$ . Assume (x,y)∈Ker{τ} , hence xτ=yτ . Therefore, xσ=x(τλ)
$$\begin{array}{c} =\left(x\;\tau \right)\lambda \;\left(\mathrm{as}\;\tau ,\lambda \;\mathrm{are}\;G-\text{acts morphism}\right)\\ =\left(y\tau \right)\lambda \\ \begin{array}{c} =y\left(\tau \lambda \right)\;\left(\mathrm{as}\;\tau ,\lambda \;\mathrm{are}\;G-\text{acts morphism}\right)\\ =y\;\sigma . \end{array} \end{array}$$

That means, (x,y)∈Ker{σ}, and so Ker{τ}⊆Ker{σ}.

⇐ ) If Ker{τ}⊆Ker{σ}, let $\varpi \;\in \;T_{{\left(F\ell \right)}_{n}\left(G\right)}$ such that ϖ : ${\left(F\ell \right)}_{n}\left(G\right)⟶{\left(F\ell \right)}_{n}\left(G\right).$ Since Im{τ}=Gxj1∪˙⋯∪˙Gxjn by, ^12^ [Lemma 5.1], define $x_{j_{ı}}\varpi$ = $y_{ı}\sigma ,$ such that $y_{ı}\tau$ = $x_{j_{ı}}$ , and $\;x_{j}\varpi =x_{j}$ , for all $j\notin \{j_{1,}j_{2,}$ …, $j_{m}\}$ .

Now, let $y_{ı}\tau$ = $\overset{´}{y_{i}}\tau =x_{j_{ı}}$ then ( $y_{ı}$ , $\overset{´}{y_{i}}$ ) ∈Ker{τ}⊆Ker{σ} means $y_{ı}\sigma =$ $\overset{´}{y_{i}}\sigma$ that implies $y_{ı}\varpi =\overset{´}{y_{i}}\varpi$ , therefore, ϖ is well –defined.

Since ${\left(F\ell \right)}_{n}\left(G\right)$ is free on $\;X_{n}=\{x_{1},\ldots .,x_{n}$ }, and as $\varpi \;\in \;T_{{\left(F\ell \right)}_{n}\left(G\right)}$ then ϖ must be a G -acts morphism. Let z∈ ${\left(F\ell \right)}_{n}\left(G\right)$ be such that $z=gx_{k},$ also let $x_{k}\tau$ = $hx_{j_{ı}}$ , that implies $\mathrm{z\tau}=\left(gx_{k}\right)\tau =g\left(x_{k}\tau \right)=g\left(hx_{j_{ı}}\right)=\mathrm{gh}\left(x_{j_{ı}}\right)=$ $\mathrm{gh}\left(y_{ı}\tau \right)=\left(\mathrm{gh}y_{ı}\right)\tau .$ Now, zτϖ = $\left(\mathrm{gh}x_{j_{i}}\right)\varpi =\left(\mathrm{gh}\right)$ ( $\;x_{j_{ı}}\varpi$ ) = $\left(\mathrm{gh}\right)\left(y_{ı}\sigma \right)=\left(\mathrm{gh}y_{ı}\right)\sigma .$

Because $\mathrm{z\tau}=\left(\mathrm{gh}y_{ı}\right)\tau ,$ and Ker{τ}⊆Ker{σ} we must have z σ = $\left(\mathrm{gh}y_{ı}\right)\sigma =\mathrm{z\tau \varpi}$ . Hence, σ=τϖ.

III. If we suppose that for any $\sigma ,\tau \;\in \;T_{{\left(F\ell \right)}_{n}\left(G\right)}$ then ℘(στ)≤℘(σ), and ℘(στ)≤℘(τ), by, ^12^ [Remark 5.2]. Recall, ℘(σ)=℘(Im{σ}) and let Im{σ}=∪˙z∈ZGz , where $Z\subseteq X_{n}$ , so that, ℘(σ)= | Z |. Because Im{στ}=Im{σ}τ=(∪˙z∈ZGz)τ=∪˙z∈ZG(zτ) this implies that ℘(στ)≤|Z|=℘(σ). Notice, ℘(τσ)=℘(Im{τσ})=℘((Im{τ})σ). As Im{τσ}⊆Im{σ} we obtain ℘(Im{τσ})≤℘(Im{σ}) and then ℘(τσ)≤℘(τ) . Therefore, ℘(στ)≤℘(σ),℘(τ). □

Theorem 6.2: For all $\;\sigma ,\tau \;\in \;T_{{\left(F\ell \right)}_{n}\left(G\right)}$ , we have the following:

1)
σLτ if and only if Im{σ}=Im{τ}.

2)
σRτ if and only if Ker{τ}=Ker{σ}.

3)
σHτ if and only if Im{σ}=Im{τ} and Ker{τ}=Ker{σ}.

4)
σDτ if and only if ℘(σ)=℘(τ) .
5)
$\sigma \;\leq_{J}\;\tau \;$ if and only if ℘(σ)≤℘(τ).

6)
σJτ if and only if ℘(σ)=℘(τ).

7)
D= J.

Proof: The point (1) and (2) can be easily verified using Theorem 6.1.

3) It is direct result from (1) and (2).

4)⟹) Let σDτ that means σRδLτ for any $\delta \;\in \;T_{{\left(F\ell \right)}_{n}\left(G\right)}$ . From (1) and (2) we get Im{δ}=Im{τ} and Ker{σ}=Ker{δ} . Now, Im{σ}≅(Fℓ)n(G)/ Ker{σ} (by the Fundamental Theorem of Semigroup), therefore, $m\left\{\sigma \right\}≅{\left(F\ell \right)}_{n}\left(G\right)/\mathrm{Ker}\;\left\{\delta \right\}$ ≅Im{δ} , and so ℘(σ)=℘(Im{σ})=℘(Im{δ})=℘(δ), and then we get ℘(σ)=℘(δ) , furthermore, ℘(δ)=℘(Im{δ})=℘(Im{τ})=℘(τ). Hence, ℘(σ)=℘(τ).

⇐) Let ℘(σ)=℘(τ). As Im{σ}=∪˙y∈YGy,Im{τ}=∪˙z∈ZGz for some $Y,Z\subseteq X_{n}$ . With | Y |=| Z | = ℘(σ)=℘(τ).

If we let φ : *Y* ⟶Ζ be a bijection and by defining ϕ:Im{σ}⟶Im{τ} by (gy ) ϕ=g(yφ), for all g∈G and y∈Y. Clearly, ϕ is one to one as where (gy ) $\phi =\left({hy}^{\prime }\right)\phi$ that implies g(yφ) = $h\left(y^{\prime }\varphi \right)$ , for all g,h∈G and $y,\overset{´}{y}\;\in \;Y$ . Then we must have g=h and (yφ) = $\left(\overset{´}{y}\varphi \right),$ this is because they are in Im{τ} and as Im{τ} is free (left)- G -act and φ is one-one, then $y=\overset{´}{y}$ . Furthermore, ϕ is onto since from definition of ϕ we get, (gy)ϕ=g(yφ) for all g∈G,y∈Y and because φ is bijection hence for all gz∈Im{τ} , choose y∈Y with yφ=z,thengy∈Im{σ}andgz=g(y)φ=(gy ) ϕ .

Suppose $\xi =\sigma \phi ,\text{where}\;\xi \;\in \;T_{{\left(F\ell \right)}_{n}\left(G\right)}$ . We have Im{ξ} = Im{σϕ} = Im{σ}ϕ=Im{τ} , therefore, τLξ.

Let $c,d\;\in \;{\left(F\ell \right)}_{n}\left(G\right).$ Clearly, cσ=dσ if and only if (cσ)ϕ=(dσ)ϕ. Since ϕ is one to one, then Ker{σ}=Ker{σϕ}=Ker{ξ} , therefore, σRξ . Hence, σDτ.

5) ⟹) Let $\sigma \;\leq_{I}\;\tau \;$ that means σ=ζτη , for any $\zeta ,\eta \;\in \;T_{{\left(F\ell \right)}_{n}\left(G\right)}$ . From Theorem 6.1, (III), we have ℘(σ)=℘(ζτη)≤℘(ζτ)≤℘(τ) .

⇐)If℘(σ)≤℘(τ),Im{σ}=∪˙y∈YGy and Im{τ}=∪˙z∈ZGz for some $Y,Ζ\subseteq X_{n}$ , with ℘(σ)= | Y | and ℘(τ)=|Z|. Because ℘(σ)≤℘(τ) then by, ^12^ [Remark 5.7] there is a one to one map ϕ:Y⟶Ζ *.* Let P=Im{ϕ} , that means P⊆Z and |Y|=|P| .

Fix $p_{0}\;\in \;P$ and define π:Z⟶P by z π=z , for all z ∈P , and $\mathrm{z\pi}=p_{0}$ for all z ∈Z\P, then Im{π}=P . Define $\;\beta :{\dot{\cup }}_{z\;\in \;Z}Gz⟶{\dot{\cup }}_{p\;\in \;P}G_{p}\;$ by zβ=zπ. Obviously, β extends to a G -acts morphism therefore, $\beta \pi \;\in \;T_{{\left(F\ell \right)}_{n}\left(G\right)}$ . Since Im{βπ} = (Im{β})π = $\left({\dot{\cup }}_{z\;\in \;Z}\mathrm{Gz}\right)\pi ={\dot{\cup }}_{z\;\in \;Z}\mathrm{Gz\pi}$ = ${\dot{\cup }}_{z\;\in \;Z}\mathrm{Gz\beta}$ = ${\dot{\cup }}_{p\;\in \;P}\mathrm{Gp}$ , we obtain ℘(βπ)=|P|=|Y|=℘(σ) so, by (4) we get βπDσ and then βπJσ because D⊆J . Therefore, $\sigma \;\leq_{I}\;\tau .$

6) ⟹) If σJτ, then $\sigma =\delta \tau \lambda ,\tau =\alpha \sigma \upsilon ,\text{for some}\;\delta ,\lambda ,\alpha ,\upsilon \;\in \;T_{{\left(F\ell \right)}_{n}\left(G\right)}.$ By Theorem 6.1, (III), ℘(σ) = ℘(δτλ)≤℘(δτ)≤℘(τ) , as well as ℘(τ)=℘(ασυ)≤℘(ασ)≤℘(σ) , that gives ℘(σ)= ℘(τ).

⇐) Let ℘(σ)= ℘(τ) , then σDτ by using (4), so that σJτ as D ⊆J .

7) This is an immediate consequence of (4) and (6).□

## 7. Conclusions

Throughout this paper, the independence algebra ${\left(F\ell \right)}_{n}\left(G\right)$ has been studied, and we show that it is free (left)- G -acts under specific conditions. Moreover, the structure of $\;T_{{\left(F\ell \right)}_{n}\left(G\right)}$ have been considered, and the we show that $\;T_{{\left(F\ell \right)}_{n}\left(G\right)}$ isomorphic to the endomorphism monoid $\mathrm{End}{\left(F\ell \right)}_{n}\left(G\right)$ , with embeddedness in the (full) wreath product of $T_{n}$ by $G^{n}$ . For future work we may consider the structure of $\;{\mathrm{PT}}_{{\left(F\ell \right)}_{n}\left(S\right)}$ , where S is a finite semigroup.

## Data Availability

Data sharing is not applicable to this article as no new data were created or analyzed in this study.
