# Supplementary material for: Structure of the (Total) Transformation Monoids Under Rank N Generators
Source: F1000Res. 2026 Apr 30;15:170. Originally published 2026 Feb 2. [Version 2] doi: 10.12688/f1000research.173831.2 (PMC13084235; doi:10.12688/f1000research.173831.2)
Supplement: Peer review history

# Reviewer report: Reviewer response for version 2

**DOI:** 10.5256/f1000research.199004.r480569

**Published:** 2026-05-13

**Authors:** Dr. Sanaa Ahmed Bajri

**Affiliations:** 1 Department of Mathematical Sciences, Princess Nourah bint Abdulrahman University, P.O. Box 84428, Riyadh, 11671, Saudi Arabia

The manuscript has been reviewed, and it appears that the authors have made the suggested amendments. I approve of this manuscript.

Is the work clearly and accurately presented and does it cite the current literature?

Yes

If applicable, is the statistical analysis and its interpretation appropriate?

Not applicable

Are all the source data underlying the results available to ensure full reproducibility?

No source data required

Is the study design appropriate and is the work technically sound?

Yes

Are the conclusions drawn adequately supported by the results?

Yes

Are sufficient details of methods and analysis provided to allow replication by others?

Yes

Reviewer Expertise:

My area is algebra and its applications.

I confirm that I have read this submission and believe that I have an appropriate level of expertise to confirm that it is of an acceptable scientific standard.

# Reviewer report: Reviewer response for version 1

**DOI:** 10.5256/f1000research.191682.r470859

**Published:** 2026-04-15

**Authors:** Katrina Belleza Fuentes

**Affiliations:** 1 University of San Carlos, Cebu City, Central Visayas, Philippines

The paper discusses the structure of transformation monoids and how they relate to endomorphisms, wreath products, and Green’s relations.

 Comments:

 1) It is weird to have sections 3,4, and 5 containing only 1 entry each: a definition. Recommend including these in the preliminaries instead and introduce each definition by a paragraph instead of having each of them in separate sections. 

 2) Definition 5.2.1 - what is the term being defined here? The writeup doesn't look like defining a term.

 3) Perhaps include an end of proof symbol to formally mark the end of a proof. 

 4) Revise format of the proof of Theorem 6.1 part II: align qualities or rewrite to a better format 

 5) I have no comments on the technicalities of the results and proof; they can be followed except that symbol used looks complicated

Is the work clearly and accurately presented and does it cite the current literature?

Yes

If applicable, is the statistical analysis and its interpretation appropriate?

Not applicable

Are all the source data underlying the results available to ensure full reproducibility?

No source data required

Is the study design appropriate and is the work technically sound?

Yes

Are the conclusions drawn adequately supported by the results?

Yes

Are sufficient details of methods and analysis provided to allow replication by others?

Yes

Reviewer Expertise:

Algebra, Algebra and Music Theory, Topological Algebra,  Algebraic Topology

I confirm that I have read this submission and believe that I have an appropriate level of expertise to confirm that it is of an acceptable scientific standard.

# Response

**Published:** 2026-04-16

**Authors:** Asawer Duraid Hamdi

The comments and suggestion made by the referee are gratefully acknowledged

 1) It is weird to have sections 3,4, and 5 containing only 1 entry each: a definition. Recommend including these in the preliminaries instead and introduce each definition by a paragraph instead of having each of them in separate sections. 

**Answer: we prefere to unchange the order of the definitons becouse we aim the reader to be connected with the context and the proof.**

 2) Definition 5.2.1 - what is the term being defined here? The writeup doesn't look like defining a term.

**Answer:  Done**

 3) Perhaps include an end of proof symbol to formally mark the end of a proof. 

**Answer:  Done**

 4) Revise format of the proof of Theorem 6.1 part II: align qualities or rewrite to a better format 

**Answer: We check the format and it is correct.**

 5) I have no comments on the technicalities of the results and proof; they can be followed except that symbol used looks complicated

**Answer: Thank you for your opinion.**

# Reviewer report: Reviewer response for version 1

**DOI:** 10.5256/f1000research.191682.r469306

**Published:** 2026-04-03

**Authors:** Dr. Sanaa Ahmed Bajri

**Affiliations:** 1 Department of Mathematical Sciences, Princess Nourah bint Abdulrahman University, P.O. Box 84428, Riyadh, 11671, Saudi Arabia

This paper addresses a study on an interesting topic in semigroup theory which is the transformation semigroups.  It provides all the required facts and properties concerning transformation semigroups. The presentation and the structure of the paper is clear and accurate. Thus, I have found it excellent paper, however, I have some minor comments throughout the paper to improve it. 

 The following are the minor comments that I have suggested:

- First Section (Preliminaries):

1. Page 3: Definition 2.2: since the action throughout the paper is on the right, the statement " α(a,b)=ab " should be written " (a,b)α =ab". 
2. Page 4: before Definition 2.6, the following parse is unnecessary " Now, let's have the following definition:" it is better to be deleted it.

- Section Five: page 5: before Theorem 5.2.2, written " the next Lemma" So, the next is it a lemma or theorem?? check this please. Also, at the same line there is a grammar mistake: It should be " in the following". 
- Section six: page 7; at the beginning of the proof of Theorem 6.2 the word "be" is repeated twice, and it should be: the point can be easily verified ...
- Throughout the paper: it will be better for the mathematical statement using the curly braces for the set to be displayed in one line.       

Is the work clearly and accurately presented and does it cite the current literature?

Yes

If applicable, is the statistical analysis and its interpretation appropriate?

Not applicable

Are all the source data underlying the results available to ensure full reproducibility?

No source data required

Is the study design appropriate and is the work technically sound?

Yes

Are the conclusions drawn adequately supported by the results?

Yes

Are sufficient details of methods and analysis provided to allow replication by others?

Yes

Reviewer Expertise:

My area is algebra and its applications.

I confirm that I have read this submission and believe that I have an appropriate level of expertise to confirm that it is of an acceptable scientific standard.

# Response

**Published:** 2026-04-16

**Authors:** Asawer Duraid Hamdi

The comments and suggestions made by the referee are gratefully acknowledged  

- First Section (Preliminaries):

1. Page 3: Definition 2.2: since the action throughout the paper is on the right, the statement " α(a,b)=ab " should be written " (a,b)α =ab". 

**Answer:  Done**

1. Page 4: before Definition 2.6, the following parse is unnecessary " Now, let's have the following definition:" it is better to be deleted it.

**Answer:  Done**

  

- Section Five: page 5: before Theorem 5.2.2, written " the next Lemma" So, the next is it a lemma or theorem?? check this please. Also, at the same line there is a grammar mistake: It should be " in the following". 

**Answer:  Done**

  

- Section six: page 7; at the beginning of the proof of Theorem 6.2 the word "be" is repeated twice, and it should be: the point can be easily verified ...

**Answer:  Done**

  

- Throughout the paper: it will be better for the mathematical statement using the curly braces for the set to be displayed in one line.

**Answer:  Done**
